# A discrete-time-evolution model to forecast progress of Covid-19 outbreak

**DOI:** 10.1371/journal.pone.0241472

**Published:** 2020-10-29

**Authors:** Evaldo M. F. Curado, Marco R. Curado

**Affiliations:** 1 Centro Brasileiro de Pesquisas Físicas, Rio de Janeiro, Brazil; 2 National Institute of Science and Technology for Complex Systems, Rio de Janeiro, Brazil; 3 AbbVie Deutschland GmbH & Co KG, Data and Statistical Sciences (DSS), Ludwigshafen am Rhein, Germany; Rey Juan Carlos University, SPAIN

## Abstract

Here we present a discrete-time-evolution model with one day interval to forecast the propagation of Covid-19. The proposed model can be easily implemented with daily updated data sets of the pandemic publicly available by distinct online sources. It has only two adjustable parameters and it predicts the evolution of the total number of infected people in a country for the next 14 days if parameters do not change during the analyzed period. The model incorporates the main aspects of the disease such as the fact that there are asymptomatic and symptomatic phases (both capable of propagating the virus), and that these phases take almost two weeks before the infected person status evolves to the next (asymptomatic becomes symptomatic or symptomatic becomes either recovered or dead). A striking advantage of the model for its implementation by the health sector is that it gives directly the number of total infected people in each day (in thousands, tens of thousands or hundred of thousands). Here, the model is tested with data from Brazil, UK and South Korea, presenting low error rates on the prediction of the evolution of the disease in all analyzed countries. We hope this model may be a useful tool to estimate the propagation of the disease.

## Introduction

A newly identified coronavirus capable of infecting humans was found in patients hospitalized in Wuhan, China [1] (and, possibly, in Europe [2]) in December 2019. Named Severe Acute Respiratory Syndrome Coronavirus-2, or SARS-CoV-2, and causing the respiratory syndrome known as Covid-19, it joins a group coronaviruses which originated from animals and resulted in human diseases, including Severe Acute Respiratory Syndrome (SARS) and Middle East Respiratory Syndrome (MERS). However, while these diseases share to some extent similar clinical symptoms, including fever, dry cough and dyspnoea [3], the Covid-19 outbreak quickly spread worldwide and became a global public health crisis. In 30 January 2020 WHO declared Covid-19 a Public Health Emergency of International Concern (PHEIC) [4], and soon thereafter, on 11 March 2020 it was declared a global pandemic. Notably, while on 30 January 2020 there were 8.324 confirmed cases worldwide, on 28 May 2020 this number increased to almost 6 million [5]. Economically, it is expected that the Covid-19 pandemic results in a recession at least as severe as the 2009 crisis [6]. As such, to reduce the social and economic burden caused by the pandemic, the development of reliable models to forecast its propagation is a promising strategy to assist public policies aiming at controlling further dissemination of the disease.

Previously published models of propagation of diseases include SIR [7], SEIR, SIS and others [8–11]. These are powerful models, as they are relatively simple and perform well on distinct infectious diseases. However, these models are in general based on differential equations and their implementation with available data are rather challenging, what makes them difficult to be widely understood and implemented. Notably, for Covid-19 the accessible data set is extensive and updated in a daily basis, including number of confirmed cases, deaths, and recovered people per country. This increases the relevance of simple models that can be easily implemented and fed with daily updated data from the pandemic, facilitating tracking of its propagation and assessment of the efficacy of public policies aiming to control it in a daily basis. Moreover, the recent history of infectious diseases have demonstrated that emergence of outbreaks are relatively frequent, to a point that such events are being included in scientific [12, 13] and political [14] discussions on preparedness for future outbreaks. Therefore, as current (in case of Covid-19) and future, yet unknown, infectious diseases will continue to impact us, the development of simple but efficient models to track pandemics evolution based on daily updated data may be valuable not only for Covid-19 but also for potential future outbreaks to come.

The data set of the Covid-19 pandemic can be found online by distinct sources, including governmental, e.g., the European Centre for Disease Prevention and Control (ECDC) [15], and non-governmental, private sources, e.g. the Johns Hopkins University [5] (JHU, from USA), and the website Worldometer [16]. These sources commonly present small differences in their data sets, but as common characteristics all present daily updated data and, among other quantities, the total number of confirmed cases of infected people and the total number of deaths per country. Here, we present a model that can easily incorporate these available data sets and is based on discrete-time equations to forecast the number of confirmed cases by Covid-19 in any given country for the next 14 days. Among its strengths, the presented model: (i) presents low relative error rates, as tested in data from Brazil, South Korea and UK; (ii) provides easily interpretable results, specifically the predicted number of infected people in the next 14 days; (iii) presents results that are directly comparable across countries; and (iv) incorporates the average time related to the disease incubation period (asymptomatic phase) and the average time related to the symptomatic phase, both parameters adjustable according to the pandemics’ characteristics.

## A discrete-time model for the Covid-19 outbreak

The main points about the Covid-19 pandemic taken into account in our model are the following:

- In a population, we can distinguish five types (classes or phases) of people. They are:
  a) people immune to the virus.
  b) people susceptible (*S*) to the virus but not infected.
  c) people infected but still in the asymptomatic phase (*A*). They can contaminate other people but present no symptoms. This phase takes roughly two weeks before clinical symptoms become apparent.
  d) people already presenting the clinical symptoms of the infection (*I*). This phase takes roughly two weeks before either recovery or death.
  e) people recovered (*R*) from the disease or dead.
- The disease follows the sequence: *S* → *A* → *I* → *R*. The time interval, *T*, going from *A* → *I* commonly vary from few days to two weeks (according to the Centers for Disease Control and Prevention, USA [17]) and we will take, for simplicity, *T* = 14 days. The time interval going from *I* → *R* can also vary from 10 to more than 20 days and again, for simplicity, we will take the same value *T* = 14 days. Clearly, this is a crude simplification since these time intervals can significantly vary per individual. For example, in patients where lungs are severely affected the recovery period is expected to take considerably longer than for patients presenting symptoms of a mild flu only. Therefore, putting an average value *T* = 14 for both phases *A* and *I* is a simplification that can only be supported by fitting with the real data set: if the fitting produces a reasonably accurate forecast, then this average value can be kept. Otherwise, the value for *T* may be fine-tuned by more sophisticated generalizations of the model. Nevertheless, in all cases we have studied, the average error rate of tested predicted values were lower than 5%.
- Immune people are not included in the model as they are assumed to not be affected by the virus, nor transmit it. Furthermore, it is assumed that a recovered person does not become susceptible again, i.e., after phase *I* the person is assumed to not be vulnerable to be re-infected by SARS-CoV-2.

We propose that the daily evolution of Covid-19 can be modelled by the following discrete-time equations:

$$\begin{array}{ccc} S(t+1) & = & S(t)-a\;S(t)\;A(t)-b\;S(t)\;I(t) \end{array}$$ (1)

$$\begin{array}{ccc} A(t+1) & = & A(t)+a\;S(t)\;A(t)+b\;S(t)\;I(t)-A(t+1-T)+A(t-T) \end{array}$$ (2)

$$\begin{array}{ccc} I(t+1) & = & I(t)+A(t+1-T)-A(t-T)-I(t+1-T)+I(t-T) \end{array}$$ (3)

$$\begin{array}{ccc} R(t+1) & = & R(t)+I(t+1-T)-I(t-T)\;, \end{array}$$ (4)

where *t* means the day, *T* means the duration of asymptomatic (incubation time) and symptomatic infected phases, both herein assumed to be 14 days. The term *aS*(*t*)*A*(*t*) indicates that a number of the susceptible people becomes asymptomatic if in contact with asymptomatic people; the same with the term *bS*(*t*)*I*(*t*) where susceptible people becomes asymptomatic if in contact with symptomatic infected people, from now on simply called infected people. The parameters *a* and *b* are constants, measuring the intensity of contagion coming from asymptomatic or infected people and having, in principle, different values. Finally, it is important to note that the sum *S*(*t*) + *A*(*t*) + *I*(*t*) + *R*(*t*) is a constant value and does not depend on time. In fact, it accounts for a fixed part of the studied population, in the order of tens of millions or eventually more for countries with large populations. As such, for these countries, the model may be described in a simplified manner if we consider that *S* is in practical terms a constant (see next section).

The parameter *T* is a key component of the model as it indicates the influence of one class into another. For example, the difference of the number of infected people from time *t* to time *t* + 1, *I*(*t* + 1) − *I*(*t*), see Eq (14), is consequence of two factors. The first one is the change in the number of asymptomatic people from time *t* − *T* to *t* + 1 − *T*, *A*(*t* + 1 − *T*) − *A*(*t* − *T*), because after a time *T* these asymptomatic people become infected people. The second one is that we have to discount the number of people leaving the class of infected ones from *t* − *T* to *t* + 1 − *T*, because they have more than 14 days since the symptoms of the disease manifested, i.e., *I*(*t* + 1 − *T*) − *I*(*t* − *T*). Hence, those become part of the class of recovered (or dead) people, *R*.

For simplicity, we have included in the same parameter, *R*, the healed and dead people. If we want to study these two cases in more detail, we can replace the Eq (4) by two equations, with the new variables *H*(*t*) (healed) and *D*(*t*) (dead) given by:

$$\begin{array}{ccc} H(t+1) & = & H(t)+(1-\mu )\;[I(t+1-T)-I(t-T)] \end{array}$$ (5)

$$\begin{array}{ccc} D(t+1) & = & D(t)+\mu \;[I(t+1-T)-I(t-T)]\;, \end{array}$$ (6)

where *R*(*t*) = *H*(*t*) + *D*(*t*) and 0 < *μ* < 1 is a parameter that indicates the percentage of infected people dead by Covid-19 after *T* days in a given country. Importantly, this parameter may vary between countries but for most countries this percentage is accessible by publicly available sources [18]. With these equations, the total number of healed and dead people can be individually predicted by the model. Here we focus on parameters *A*(*t*) and *I*(*t*), but in future works these two other variables will be analyzed.

## A simplified discrete-time model

The model presented in the last section can still be simplified for countries with large population. First, as remarked before, it should be noted that the number of susceptible people can be a large number for countries with millions of inhabitants. The epidemic starts with a very small number of infected people, that increases and can reach (as currently seen) hundreds of thousands of people. For countries with large population, e.g., several tens (or even more than a hundred) million habitants, we can further simplify the model described in Eqs (1)–(4) assuming that the number of susceptible people is practically constant in time, *S*(*t*) ≃ *S*, where *S* is a constant, so there is no time evolution for *S*. This can be assumed for countries with large populations as the difference between infected (asymptomatic *A* + symptomatic *I*, which theoretically may reach up to few hundred thousand people in a given country) and susceptible people *S* (depending on the country reaching over a hundred million people) is still so huge that the decrease in the sample of susceptible people *S* may be negligible. As such, calling 1 + *aS* ≡ *α* and *bS* ≡ *β*, where *α* and *β* are positive constants, the equations for Covid-19 for large-population countries can be written as:

$$\begin{array}{ccc} A(t+1) & = & \alpha \;A(t)+\beta \;I(t)-A(t+1-T)+A(t-T) \end{array}$$ (7)

$$\begin{array}{ccc} I(t+1) & = & I(t)+A(t+1-T)-A(t-T)-I(t+1-T)+I(t-T) \end{array}$$ (8)

$$\begin{array}{ccc} R(t+1) & = & R(t)+I(t+1-T)-I(t-T)\;, \end{array}$$ (9)

where *A*, *I* and *R* are counted by hundreds, thousands or hundred of thousands. Hence, here the sum *A* + *I* + *R* is not a constant anymore, but an increasing function on time. The susceptible people are, in these cases, considered as a ‘thermostat’, practically not changing in time. Notice that here the total number of inhabitants of a country is not an important parameter, as it is in other disease models.

This simplified model can be used while the number of *A* + *I* + *R* is lower than a small percentage of the total number of susceptible people of that country. Conservatively, we recommend to use it while this sum is lower than 5%. However, this threshold of 5% is not strict and may be modified to some extent, but if the number of *A* + *I* + *R* is larger than 10% of susceptible people, the accuracy of the simplified version of the model may be affected. In these cases, the complete model represents a better alternative to forecast progress of Covid-19.

An analysis of these equations shows that Eqs (15) and (16) are the key equations of the model, with the Eq (9) being a supportive equation. This system of equations has only two parameters, *α* and *β*, that can be found phenomenologically for each country and roughly measures the capacity of contagion of asymptomatic and symptomatic people. This model could have more parameters, but it seems that all other parameters can be condensed in these two, with the only requirement of having positive values.

## How to use available data on this model

The data set provided by the above-mentioned sources are updated in a daily basis and have at least two useful information for the proposed model: the number of deaths and the total number of confirmed cases (total symptomatic infected) people, *I*_*tot*_(*t*) per country and globally. The main variables in our model are the number of infected (i.e., asymptomatic *A*(*t*) + symptomatic *I*(*t*)) people at time *t*, i.e. people that at time *t* can infect susceptible people. There are many definitions of symptomatic people in these sources, and they are not completely equivalent. For our model, we will define the class of symptomatic people by means of the total number of confirmed cases by country, as the definition of confirmed cases is the same on all sources.

With our hypothesis we can simply define:

$$\begin{array}{c} I\left(t\right)=I_{tot}\left(t\right)-I_{tot}\left(t-T\right)\;, \end{array}$$ (10)

that is, the number of infected people at time *t*, defined as *I*(*t*), is the total number of infected people at time *t* minus the total number of infected people at time *t* − *T*. In turn, the total number of infected people at time *t* − *T* represents people who either recovered or died after the infection, since their symptoms manifested in more than *T* days. Hence, by defining *t*_0_ as the first day when someone is identified with the symptoms of the infection in a given country, *I*(*t*_0_) as the “first infected people”, i.e., the number of people infected at day *t*_0_, and *t* = *t*_*N*_ as the last day when data is collected, we can construct the corresponding data set of the variable *I*(*t*) from *t* = *t*_0_ until *t* = *t*_*N*_. Here, we are assuming that *I*_*tot*_(*t*_0_ − *τ*), where *τ* is a positive integer, is equal to zero since we are considering *t*_0_ as the day of the first infected people (with symptoms). This ensues that *I*(*t*) = 0 for 1 ≤ *t* < *t*_0_. In fact, in our model, we always have *t*_0_ = *T* + 1 = 15. Our day one for a specific country is always *t*_0_ − *T*, the day of the first asymptomatic people.

Now, we can construct, from the recent built *I*(*t*) time series, the time series of the other variable, *A*(*t*). For that we use Eq (16) rewritten as,

$$\begin{array}{c} A(t+1)-A(t)=I(t+T+1)-I(t+T)+I(t+1)-I(t)\;. \end{array}$$ (11)

With this equation we can construct the time series of *A*(*t*) from *t* = *t*_0_ − *T* = 1 until *t* = *t*_*N*_ − *T*. As *t*_0_ − *T* = 1, i.e., the first day when people got contaminated (but still asymptomatic), we can define the initial condition in order to solve Eq (11),

$$\begin{array}{c} A\left(t_{0}-T\right)=A\left(1\right)=I\left(t_{0}\right)=I_{tot}\left(t_{0}\right)\;. \end{array}$$ (12)

After *T* days these people will lead to the first infected people, at time *t* = *t*_0_ = *T* + 1. So, from the real data set of total infected people we can construct the time series of *I*(*t*) from *t* = 1, ⋯, *t*_*N*_ and, from Eqs (11) and (12), the time series of *A*(*t*) from *t* = 1, ⋯, *t*_*N*_ − *T*.

In the next step we use Eqs (15) and (16) to generate the *theoretical* time series for *A*(*t*), from *t* = *t*_*N*_ − *T* + 1, and for *I*(*t*) from *t*_*N*_ + 1. For that we need the values of *α* and *β*.

### Obtaining adequate values for *α* and *β*

We need the series of *A*(*t*) and *I*(*t*) to forecast the next *T* days for the total number of infected people starting on day *t*_*N*_. One way to try to get good values for the parameters *α* and *β* is to make the prediction using Eqs (15) and (16) not from *t*_*N*_ but, say, from *t*_*N*_ − 5 until *t*_*N*_, that is, we consider the real data until the day *t*_*N*_ − 5 for *I*(*t*) and the real data until *t*_*N*_ − *T* − 5 for *A*(*t*). This way it is possible to check the theoretical values of *A*(*t*), from *t*_*N*_ − *T* − 5 until *t*_*N*_ − *T*, *I*(*t*) and *I*_*tot*_(*t*) from *t*_*N*_ − 5 until *t*_*N*_ with the real ones, obtained from the real data set until *t*_*N*_. Adjusting *α* and *β* to best fit these, say, five values, the forecast of the values of *A*(*t*), *I*(*t*) and *I*_*tot*_(*t*) from *t*_*N*_ until *t*_*N*_ + *T* + 1 ensue. In fact, the prediction for *A*(*t*) and *I*(*t*) has no upper bound for the chosen values of *α* and *β*, only the prediction for *I*_*tot*_(*t*) is limited to the next *T* days due to Eq (10) if we want to keep using real data. Other ways to obtain good values for the parameters *α* and *β* can be conceived, but we propose this simple way as it worked well in the tested cases.

The values of *α* and *β* can change over time following changes on social behaviour, e.g. due to measures of confinement by local governments or increasing consciousness of the population, which in turn affect the interactions of susceptible people with asymptomatic and symptomatic infected people. Therefore, it is recommendable to test the adequacy of the parameters *α* and *β* along the progression of the disease. In the next section we will see examples of the application of the model in some countries.

## Case studies

If we regard the graphs of total infected people for several countries, we can notice three standard patterns. The first one shows a curve increasing faster than a linear one. These are the cases of Brazil, Russia, India, among others. The second pattern shows an almost linear increasing curve, e.g., UK and US. Finally, the third one presents a strong increasing in the beginning and then the curve tends to saturate, e.g., South Korea, Germany and China. We will discuss examples of each one of these three patterns. In all cases we are considering the data until May 28th. Then, we use the days from May 23th or 24th until May 28th to adjust good values for *α* and *β*. These values can then be used for prediction from May 28th until June 11th.

### Brazil

**Fig 1:**
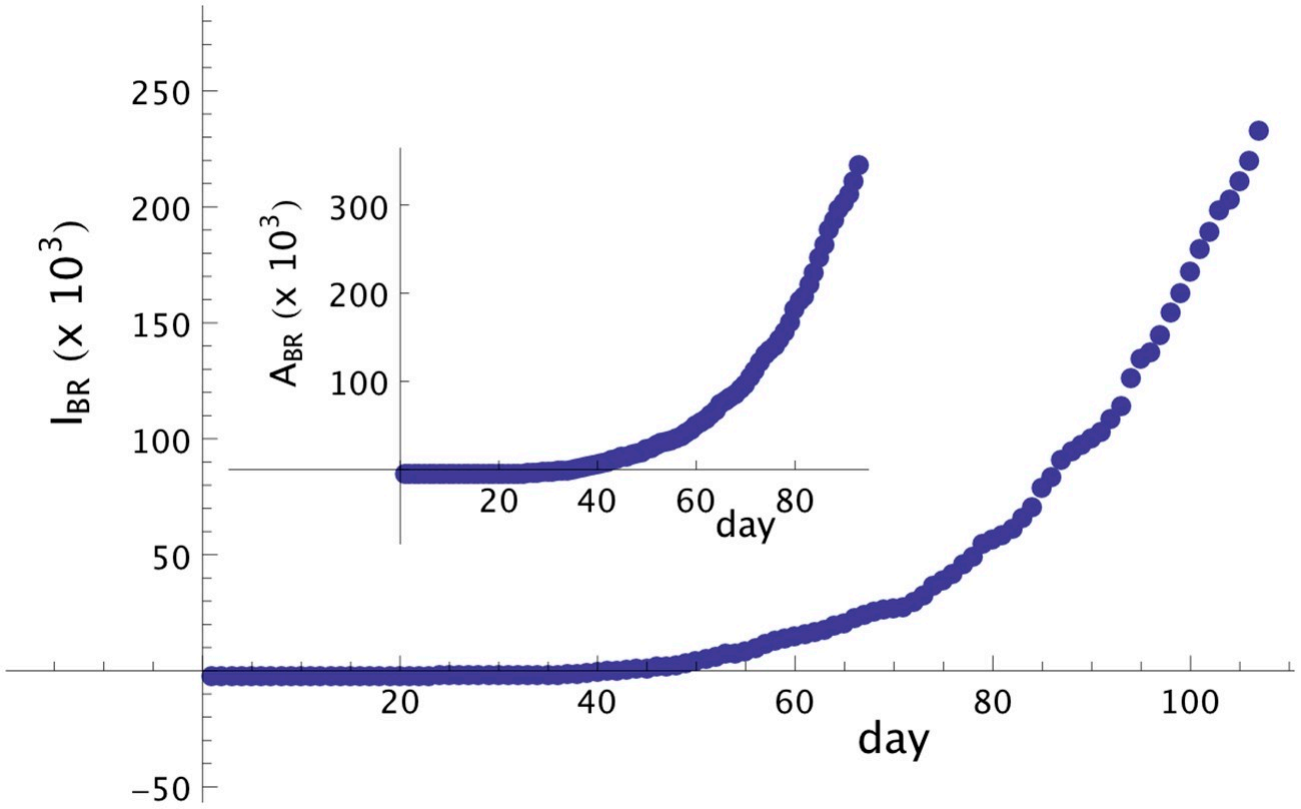
Symptomatic and asymptomatic cases of Covid-19 in Brazil. Number of symptomatic infected people in Brazil from day 01, February 12th, until day 107, May 28th. Notice that the curve for symptomatic infected people presents an increasing monotonic behavior. In the inset we show the curve of asymptomatic (but infected) people until day 93, that also presents a strong monotonically increasing behavior.

The first infected person in Brazil was identified on February 26th, defined here as day *t*_0_. Using *T* = 14, the first infected person according to our model was identified on February 12th (i.e., 14 days before), defined here as day *t* = 1. The time series of the total infected people from the day 01, February 12th, until May 28th (107 days in total), can be obtained from the sources [5, 15, 16]. We are using herein, in this case, the data set from [5]. Based on this data set we can construct the time series of *I*_*BR*_(*t*) from *t* = 1, ⋯, *t*_*N*_ = 107 and *A*_*BR*_(*t*) from *t* = 1, ⋯, *t*_*N*_ − 14 = 93 (Fig 1). Here, we can implement our proposed model to predict 14 days after day 107.

With the intention to test the method and find good values for the parameters *α* and *β*, we considered our fictitious $t_{N}^{f}=102$-th day, so it is possible to compare the total number of infected people as provided by the original source (JHU) with the predicted data generated by the model. For this comparison we will focus on days 103 to 107 and we consider only the real data of *I*_*BR*_(*t*) up to day 102 and data of *A*_*BR*_(*t*) up to day 88. Adjusting the predicted data generated by the method with the real data values, we found that for *α* = 1.01 and *β* = 0.17 the relative error from days 103 to 107 is lower than 2%. The relative error in this period of five days is less than 2%.

**Fig 2:**
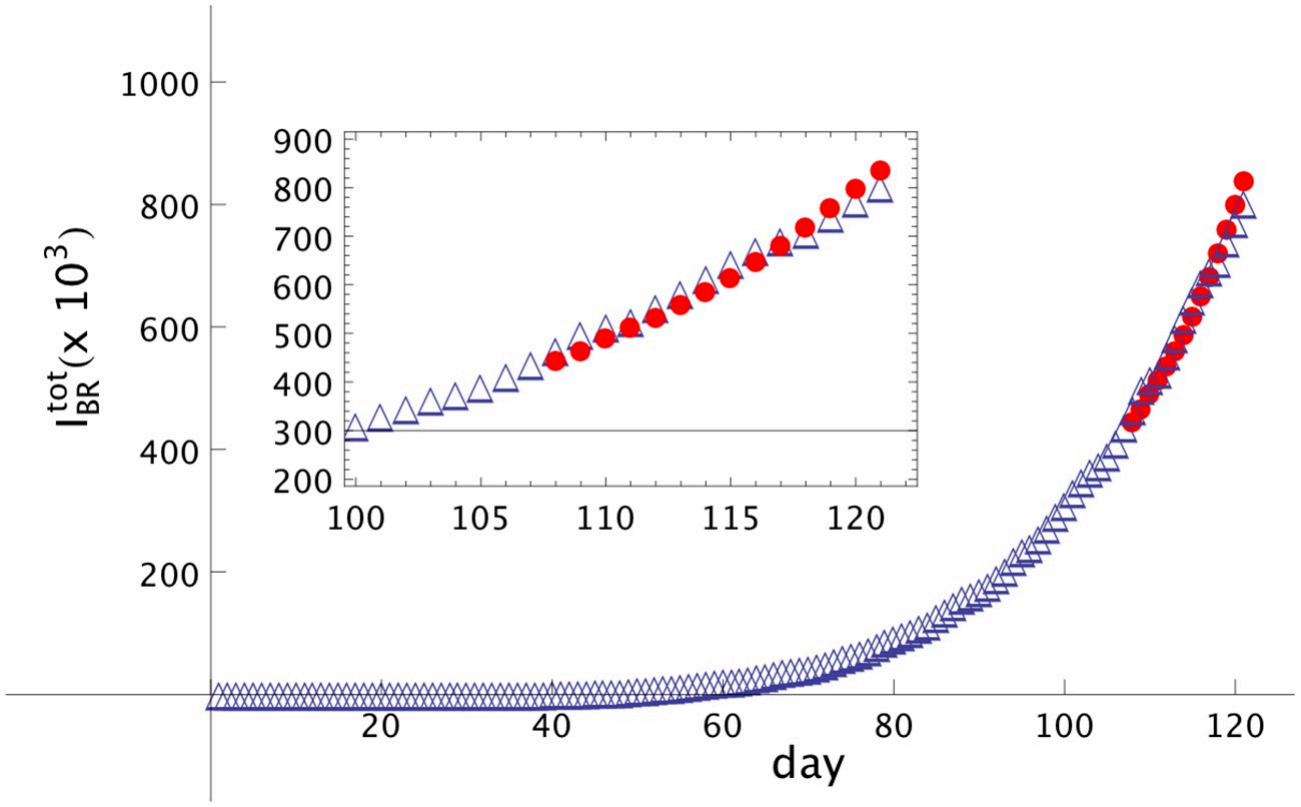
Modelling of Covid-19 dissemination in Brazil. Total number of people infected by Covid-19 in Brazil, $I_{BR}^{tot}$, as function of the number of days, starting from February 12th, indicated as number 1. Empty triangles (red) are real data and full circles (red) are the forecast from day 107, May 28th, until day 121, June 11th. *α* = 1.01 and *β* = 0.17. Notice that the curve increases faster than a linear one. In the inset we show a shorter interval, from day 100 up to day 121.

These values can then be applied to estimate the total number of infected people *T* = 14 days following day 107 (Fig 2). The empty triangles (red) are the real data obtained from [5] until day 121, June 11th. The full circles (red) are the numbers predicted by the model starting from day 108 and based on the real data until day 107. The maximal relative error, defined as $\text{error}(\text{t})=(I^{tot}(t)-I_{model}^{tot}(t))/(I^{tot}(t))$ registered on the last day of the forecast, was 5.27%. *I*^*tot*^(*t*) is the total registered number of infected people in a specific country and $I_{model}^{tot}\left(t\right)$ is the total number of infected people given by the numerical simulation of the model, at day *t*.

The inset shows a shorter scale, from day 100 until day 121.

It should be noted that in the case of Brazil the values for parameters *α* and *β* are practically constant from April to early May, but from May to June it is possible to notice a slight decrease on these values. This does not happen with the other countries here presented, where the values of the parameters decrease faster with time. Accordingly, the parameters *α* and *β* should be updated for each change on the monotony of the curve of infected people, since these changes may reflect new social behaviours affecting the contagion parameters *α* and *β*, with consequences on the time evolution of the disease. Clearly, these changes are unpredictable because they depend on public health polices and population awareness.

### UK

**Fig 3:**
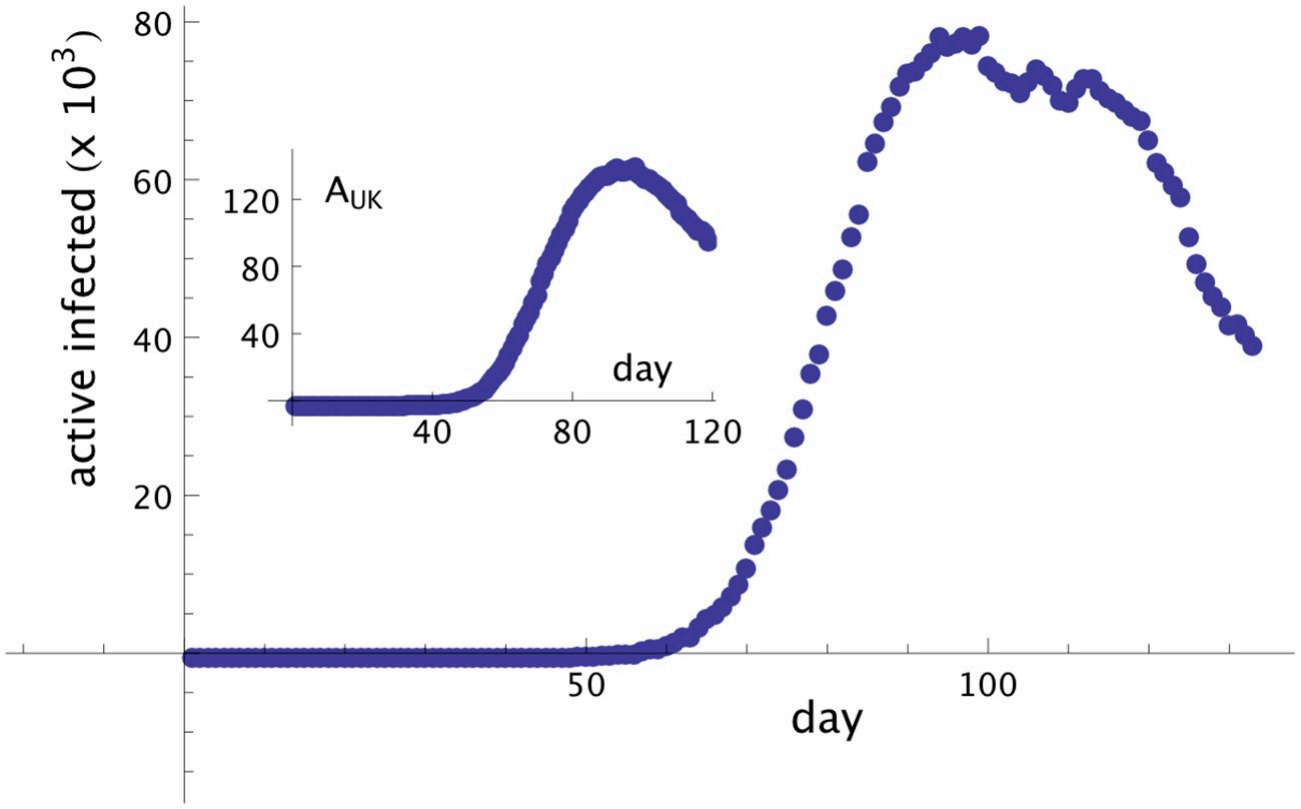
Symptomatic and asymptomatic cases of Covid-19 in UK. Number of infected, symptomatic people in UK, *I*_*UK*_, from January 17th until May 28th, day 133. In the inset, the asymptomatic (but infected) people, *A*_*UK*_, in thousands and until day 119, is shown.

Here, we consider the real data of the total number of infected people by Covid-19 in UK. The first people identified in UK presenting symptoms of Covid-19 were registered on January 31th, which we define here as day *t*_0_. Using *T* = 14, the date of contagion for those people was on January 17th, defined here as day *t* = 1. The data set of the total infected people from the day 01, January 17th, until May 28th (133 days in total), can be obtained from the sites [5, 15, 16]. Here we are using the data set from the Johns Hopkins University [5]. From this data set we can build the time series of symptomatic infected people ${\left\{I_{UK}\left(t\right)\right\}}_{t=1}^{133}$, and asymptomatic infected people ${\left\{A_{UK}\left(t\right)\right\}}_{t=1}^{119}$ (Fig 3). In UK, the curves for symptomatic infected people and asymptomatic people suggest that the peak of the contagion ended roughly 100 days after the first registered case of infection. Besides that, some oscillations can be found following the peak and prior to the pronounced decline in contagion. This is in contrast with the curve profiles from Brazil, as there they are monotonically increasing over time. Here, in order to capture the oscillatory profile from UK into the model and estimate good values for parameters *α* and *β*, it is better to consider a small interval of time for the test, with all data points belonging to the slope of the new tendency.

**Fig 4:**
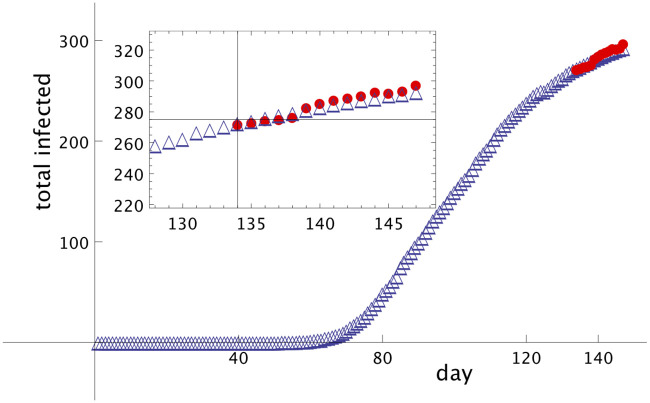
Modelling of Covid-19 dissemination in UK. Total number of Covid-19 infected people in UK as function of the number of days, starting from January 17th, marked as number 1. Empty triangles (blue) are real data up to day 147 (June 11th), and full circles (red) are the prediction of the model starting on day 134 (May 29th) until day 147 (June 11th). *α* = 0.94 and *β* = 0.001. The inset shows a short scale, from day 128 up to day 147. The forecast (full circles, red) was made based on day 133 and previous days.

Therefore, to test the method in UK, we will consider our fictitious *t*_*N*_ = 129-th day, four days less the true *t*_*N*_ = 133. As a consequence, it will be possible to test the days from 129 until 133, generated by the method (for this test we are considering only the real data of *I*_*UK*_(*t*) up to the day 129 and of *A*_*UK*_(*t*) up to day 115). The agreement among the real data and the points predicted by the method from day 129 until day 133 is quite good for *α* = 0.94 and *β* = 0.001. Using these values for the parameters we can predict the evolution of the total number of infected people in UK from day 133 until day 147 (June 11th). Fig 4 shows the real data, with empty triangles (blue), and the numbers predicted by the method, with full circles (red), for *α* = 0.94 and *β* = 0.001. The inset shows a window from day 128 until day 147. The agreement is quite good for 14 days and the maximum error in these period is always smaller than 4%.

### South Korea

**Fig 5:**
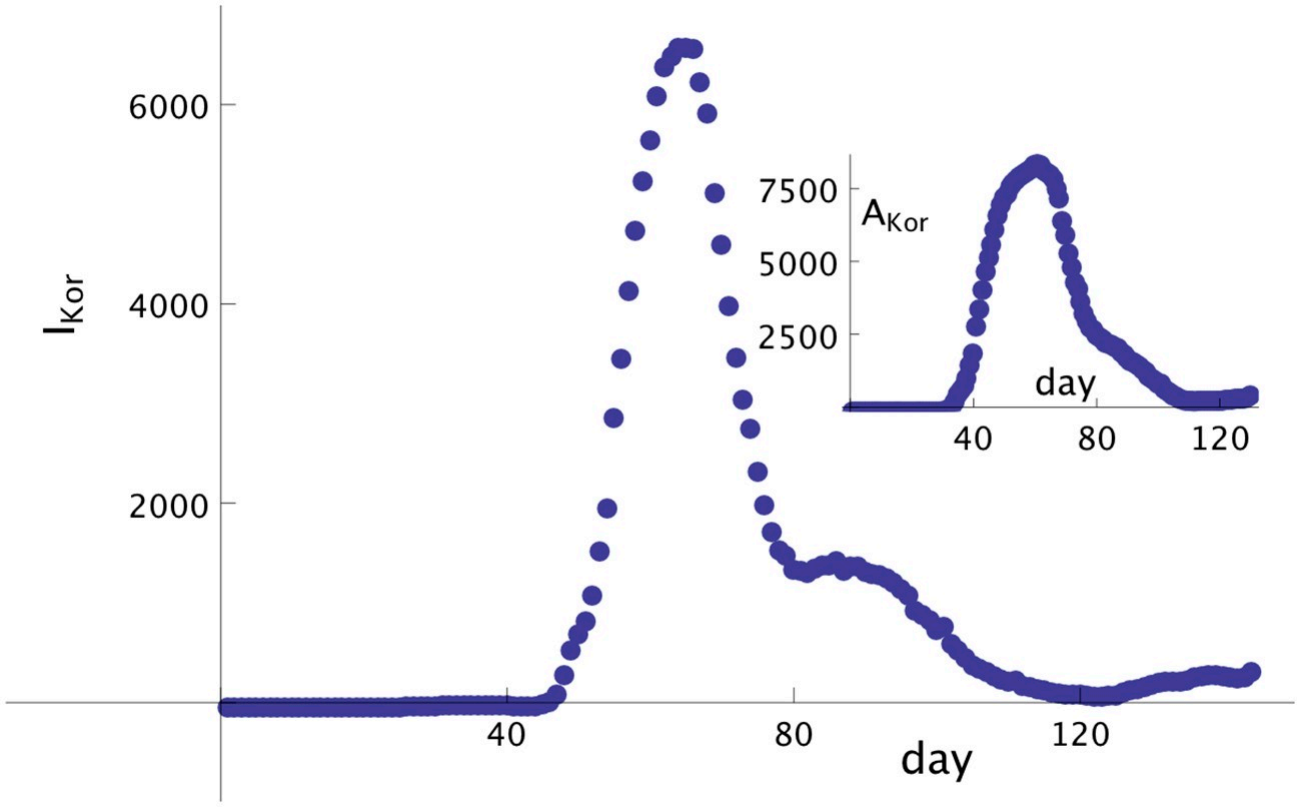
Symptomatic and asymptomatic cases of Covid-19 in South Korea. Infected (symptomatic) (*I*_*Kor*_) people until day 144 (May 28th) in South Korea. Inset: asymptomatic (but infected) (*A*_*Kor*_) people until day 130 (May 14th). The propagation of the disease is controlled but some oscillations still remain.

**Fig 6:**
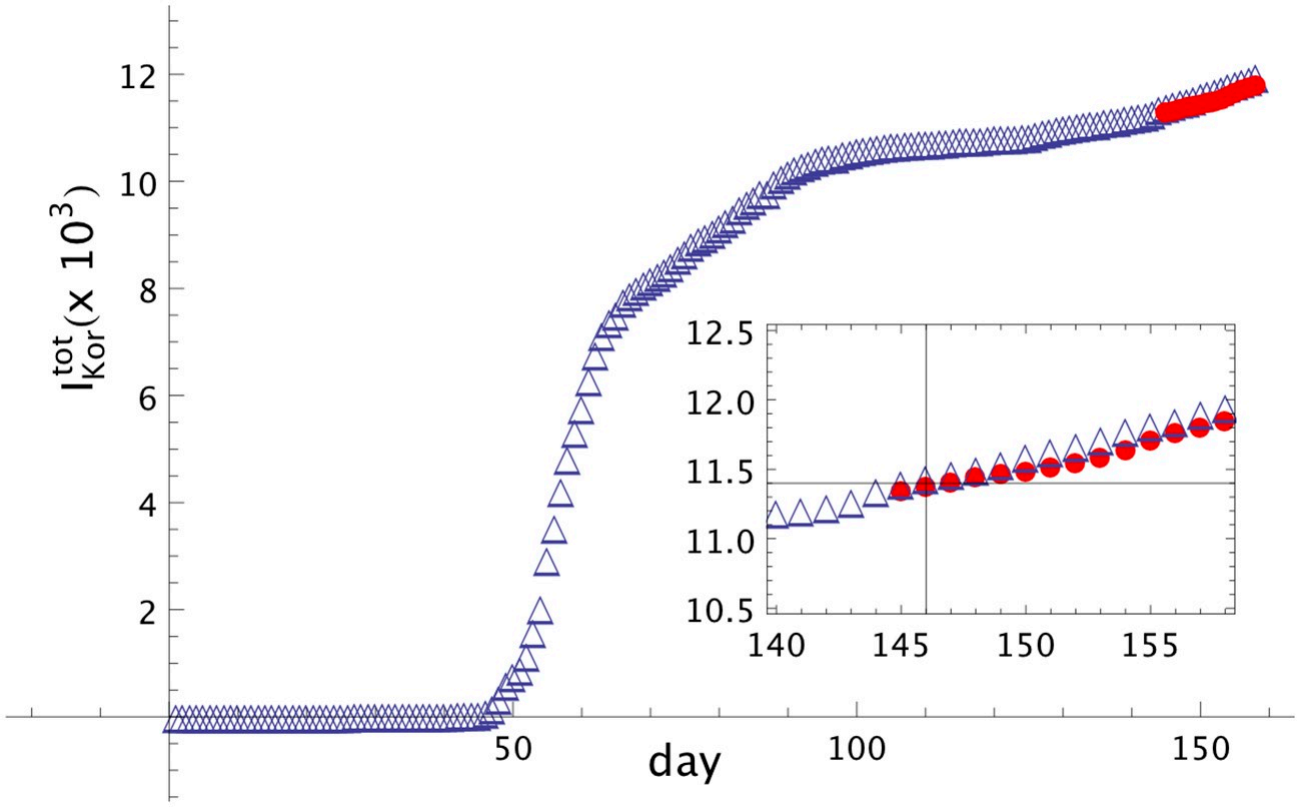
Modelling of Covid-19 dissemination in South Korea. Total infected people in South Korea, ($I_{Kor}^{tot}\left(t\right)$), until day 158 (June 11th). Empty triangles (blue) are real data from [15]. Full circles are the forecast, using the values *α* = 1.04 and *β* = 0.02, from day 144 until day 158 (June 11th). In the inset the window from day 140 until day 158 is shown, in thousands.

The first infected person with symptoms in South Korea was identified on January 20th, our day *t*_0_. In our model this means that the first asymptomatic person was infected on January 6th, here defined as day 1. The data set from January 06 (day 1) until May 28th (day 144), the last day where data was considered for forecast, was obtained from the ECDC site [15] (i.e. demonstrating the implementation of the model based on data set from a distinct source). Based on the data of total infected people, we can build the time series for the (symptomatic) infected people, ${\left\{I_{Kor}\left(t\right)\right\}}_{1}^{144}$, see Eq (10). From the time series of {*I*_*Kor*_(*t*)}, and using Eqs (11) and (12), we can build the time series for the asymptomatic people, ${\left\{A_{Kor}\left(t\right)\right\}}_{1}^{130}$. Fig 5 shows the graph of infected and asymptomatic (inset) people in South Korea in each day. In Fig 6 it is shown the real total number of infected people in South Korea until day 158, June 11th, with (blue) empty triangles. The full circles (in red) are the prediction of the model, keeping *α* = 1.04 and *β* = 0.02 constants, until day 158, June 11th. The error in the whole period is smaller than 0.85%, demonstrating remarkable accuracy.

## Discussion

In the present study we build and present a discrete-time model to study the time evolution of the Covid-19 pandemic based mainly on the numbers of asymptomatic and symptomatic infected people. The model has only two parameters, *α* and *β*, what facilitates its implementation by health sector personnel and interested people in general. It is possible to use the daily updated data set of total confirmed cases of infected people to choose values for these parameters and predict how this number of infected people changes in the next 14 days if the parameters are kept constant. The model gives the effective numbers of total infected people, in tens or hundreds of thousands, that can be directly compared with the data set available from distinct sources, including Johns Hopkins University, ECDC, Worldometer and others. Also, the model incorporates the average time-delay asymptomatic people take to become symptomatic (incubation period) and the average time-delay the symptomatic infected people take to recover or, eventually, die. Here, the model has been implemented in three countries (Brazil, UK and South Korea), and the predicted values generated by the model from May 29th until June 11th, based on the real data set until day May 28th, agrees very well with the real numbers of total infected people during this period. The maximum daily error for these three cases, each one with a period of 14 days, is around 5%. As such, we have found that the model is able to provide a reliable estimate of the status of the disease at least two weeks ahead of analysis time. Remarkably, this is achieved with a rather simple algorithm, based only on fitting two parameters and on publicly available data [5, 15, 16]. Altogether, we believe these characteristics facilitate its implementation by public sector, and may be a useful tool to track disease propagation and efficacy of public policies aiming to control it.

It may be argued that the model is over-simplistic and do not account for complexities from the pandemic evolution. For instance, it has been reported that the coronavirus can still be detected in throat swabs from recovered patients up to 13 days after they present no symptoms of the disease (as evaluated by clinical assessments), indicating that even people considered to be recovered from Covid-19 may still potentially infect others [19]. Furthermore, it has been speculated the possibility of reinfection for patients who recovered from Covid-19 (in this regard, preliminary data from primates support the hypothesis of immunization and protection against reinfection [20]). In fact, we are aware that the model does not cover extensively the possible complexities related to disease propagation, but it focuses on the main course of propagation instead. As the model indicates a small error rate in its prediction, we considered it to fulfil its purpose adequately, exchanging complexity for simplicity in its comprehension and implementation while maintaining satisfactory predictive power (accuracy). These, in our understanding, are core principles for its assimilation by public health sector.

Moreover, the assessment of some complexities related to the pandemic may be heavily influenced by regional factors, what makes them challenging to reliably model, interpret or compare results between countries. For instance, some patients may develop from asymptomatic phase *A* to recovered phase *R* without undergoing the symptomatic phase *I*, a situation not included in the model. Here, a challenge for modelling those cases is that not only the number of Covid-19 testing varies dramatically between countries but also the infectious status of the tested population may vary significantly between countries. For example, it is expected that countries testing the population more extensively are detecting more asymptomatic subjects (including those in the above-mentioned scenario), while countries with less testing are prioritizing diagnosing symptomatic patients, what risks biasing the model towards countries with more data available. Still, having discussed the challenges related to reliably assessing these cases, one could evaluate their effects in our model by adding a new parameter *γ* in Eqs (2) and (3), and modifying them into the equations:

$$\begin{array}{ccc} A(t+1) & = & A(t)+a\;S(t)\;A(t)+b\;S(t)\;I(t)-\gamma \;[A(t+1-T)-A(t-T)] \end{array}$$ (13)

$$\begin{array}{ccc} I(t+1) & = & I(t)+\gamma \;[A(t+1-T)-A(t-T)]-I(t+1-T)+I(t-T)\;, \end{array}$$ (14)

implying the changes in Eqs (5) and (6):

$$\begin{array}{ccc} A(t+1) & = & \alpha \;A(t)+\beta \;I(t)-\gamma \;[A(t+1-T)-A(t-T)] \end{array}$$ (15)

$$\begin{array}{ccc} I(t+1) & = & I(t)+\gamma \;[A(t+1-T)-A(t-T)]-I(t+1-T)+I(t-T)\;, \end{array}$$ (16)

where 0 < *γ* < 1. This means that not all asymptomatic people between days *t* − *T* and *t* + 1 − *T* will become infected people *T* days after. It should be emphasized, though, that to explicitly include these cases into the model comes at the cost of (a) increasing the complexity of the model by introducing another parameter, *γ*, (b) risking accuracy, as this information is challenging to reliably assess in any country (unless massive testing is implemented in the population in a narrow time window), and (c) potentially impacting the comparability across countries, as the assessment of this population may be largely different between countries, as discussed above. In the simplified model (i.e., without parameter *γ*), we assume that this parameter is integrated in the parameter *α*, decreasing its value, and consequently reducing the number of infected people without increasing the number of parameters. Considering the good predictive power presented by the simplified model, we believe that its simplistic approach based on the main course of the pandemic has the advantages of being easier to implement, and providing more standardized, interpretable and comparable results across countries.

We would like to remark that the prediction for the total number of infected people is restricted to 14 days because of the definition given in Eq (10), where we want to use the real data for the total number of infected people. If we relax this requirement to use real data, we can get points for the whole future, keeping the parameters constant. Certainly, this is not expected and this prediction for a long time is likely not reliable. It only gives an estimate of how will the disease propagate in the future if a given country does not change the value of the parameters. In fact, in practically all tested cases the parameters *α* and *β* decreased on time, likely reflecting measures against the propagation of the disease. For this reason, predictions provided by the model here presented are expected to decrease its accuracy for longer periods.

The people from phase *R* incorporates those who recovered as well as those who died following the infection. Thus, it is also possible to estimate separately the number of recovered and the number of dead people in two weeks, by simply applying in the *R* estimate the percentage of recovered and dead people from the studied country. As this is straightforward, we did not analyze this aspect in this study, but it is planned to be done in future works.

A last word about the model: it is noteworthy, the model may also be used in other similar diseases—having both asymptomatic and symptomatic phases and both being contagious—as long as these other diseases have a daily (or some periodic) update of the data set. Clearly, the parameter *T* has to be adapted to the life cycle of the studied pathogen and it also is plausible to require two distinct *T*′’s, one for the asymptomatic and other for the symptomatic phase. This, however, needs to be fitted to the specific characteristics of the studied disease.

Finally, a more extensive work is being prepared, with the analysis of many other countries and a study of the parameter space. We hope the present model can contribute to global efforts made to understand and control the Covid-19 pandemic.

## References

[1] Wei-jie G, Zheng-yi N, Yu H, Wen-hua L, Chun-quan O, Jian-xing H, et al Clinical characteristics of coronavirus disease 2019 in China. New Eng J of Med. 2020; 382 (18): 1708–1720. 10.1056/NEJMoa2002032 32109013 PMC7092819

[2] Deslandes A, Berti V, Tandjaoui-Lambotte Y, Chakib A, Carbonnelle E, Zahar JR, et al SARS-COV-2 was already spreading in France in late December 2019. Intern J of Antimicrob Ag. 2020; 106006 10.1016/j.ijantimicag.2020.106006 32371096 PMC7196402

[3] Chaolin H, Yeming W, Xingwang L, Lili R, Jianping Z, Yi H, et al Clinical features of patients infected with 2019 novel coronavirus in Wuhan, China. The Lancet. 2020; 395: 497–506. 10.1016/S0140-6736(20)30183-5 PMC7159299 31986264

[4] World Health Organization and others *COVID 19 Public Health Emergency of International Concern (PHEIC)* *Global research and innovation forum: towards a research roadmap*. 2020.

[5] Johns Hopkins University: Coronavirus Research Center. Available from: https://coronavirus.jhu.edu/map.html

[6] United Nations. Shared responsibility, global solidarity: Responding to the socio-economic impacts of COVID-19. 2020.

[7] Kermack WO, McKendrick AG. A Contribution to the Mathematical Theory of Epidemics. Proc of the Royal Soc of London. Series A: Mathematical, Physical and Engineering Sciences. 1927; 115 (772): 700–721.

[8] Brauer F, Castillo-Chavez C. Mathematical models in Population Biology and Epidemiology 2nd ed New York: Springer; 2010.

[9] Keeling MJ, Rohani P. Modeling Infectious Diseases in Humans and Animals. Princeton: Princeton University Press; 2008.

[10] Manotosh M, Soovoojeet J, Swapan Kumar N, Anupam K, Sayani A, Kar TK. A model based study on the dynamics of COVID-19: Prediction and control. Chaos, Solit & Fract. 2020; 109889. 10.1016/j.chaos.2020.109889 PMC7218394 32406395

[11] Tsallis C, Tirnakli U. Predicting COVID-19 peaks around the world. Front in Phys. 2020; 8: 217 10.3389/fphy.2020.00217

[12] Anne S, Beth PB, Stephen CR. The science behind preparing and responding to pandemic influenza: the lessons and limits of science. Clin Infect Dis. 2011; 52: S8–S12. 10.1093/cid/ciq007 21342904

[13] Kouadio K, Okeibunor J, Nsubuga P, Mihigo R, Mkanda P. Polio infrastructure strengthened disease outbreak preparedness and response in the WHO African Region. Vaccine. 2016; 34: 5175–5180. 10.1016/j.vaccine.2016.05.070 27378681

[14] Paules CI, Eisinger RW, Marston HD, Fauci AS. What Recent History Has Taught Us About Responding to Emerging Infectious Disease Threats. Ann of Int Med. 2017; 167: 805–811. 10.7326/M17-2496 29132162

[15] European Centre for Disease Prevention and Control (ECDC). Available from: https://www.ecdc.europa.eu/en/covid-19-pandemic

[16] Worldometer: COVID-19 CORONAVIRUS PANDEMIC. Available from: https://www.worldometers.info/coronavirus

[17] Centers for Disease Control and Prevention (CDC): Coronavirus Disease 2019 (COVID-2019). Available from: https://www.cdc.gov/coronavirus/2019-ncov/symptoms-testing/symptoms.html

[18] Johns Hopkins University: Coronavirus Research Center. Mortality Analysis. Available from: https://coronavirus.jhu.edu/data/mortality.

[19] Lan L, Dan X, Guangming Y, Chen X, Shaokang W, Yirong L, et al Positive RT-PCR test results in patients recovered from COVID-19. JAMA. 2020; 323: 1502–1503. 10.1001/jama.2020.2783 32105304 PMC7047852

[20] Ota M. Will we see protection or reinfection in COVID-19? Nat Rev Immunol. 2020; 20:351 10.1038/s41577-020-0316-3 32303697 PMC7186928

